# Green Synthesis of Antileishmanial and Antifungal Silver Nanoparticles Using Corn Cob Xylan as a Reducing and Stabilizing Agent

**DOI:** 10.3390/biom10091235

**Published:** 2020-08-25

**Authors:** Rony Lucas Silva Viana, Gabriel Pereira Fidelis, Mayara Jane Campos Medeiros, Marcelo Antonio Morgano, Monique Gabriela Chagas Faustino Alves, Luiz Felipe Domingues Passero, Daniel Lima Pontes, Raquel Cordeiro Theodoro, Thales Domingos Arantes, Diego Araujo Sabry, Guilherme Lanzi Sassaki, Raniere Fagundes Melo-Silveira, Hugo Alexandre Oliveira Rocha

**Affiliations:** 1 Departamento de Bioquímica, Universidade Federal do Rio Grande do Norte, Natal, RN, Rio Grande do Norte 59078-970, Brazil; rony_lucas@hotmail.com (R.L.S.V.); gabrielfideliss@gmail.com (G.P.F.); monique.gabi@gmail.com (M.G.C.F.A.); raquel.ctheodoro@gmail.com (R.C.T.); smah05@hotmail.com (T.D.A.); popoh.diego@gmail.com (D.A.S.); ranierefagundes@hotmail.com (R.F.M.-S.); 2 Departamento de Química, Universidade Federal do Rio Grande do Norte, Natal, RN, Rio Grande do Norte 59078-970, Brazil; mayarajane20049@hotmail.com (M.J.C.M.); pontesdl@yahoo.com (D.L.P.); 3 Centro de Ciências e Qualidade dos Alimentos (CCQA), Instituto de Tecnologia dos Alimentos (ITAL), Campinas 13070-178, SP, Brazil; morgano@ital.sp.gov.br; 4 Instituto de Biociências, Universidade Estadual de São Paulo (Unesp), Praça Infante Dom Henrique, s/n, São Vicente 11330-900, SP, Brazil; felipepassero@clp.unesp.br; 5 Departamento de Bioquímica, Universidade Federal do Paraná, Curitiba 80060-000, PR, Brazil; sassaki@ufpr.br

**Keywords:** polysaccharides, antiparasitic, agricultural byproduct, nanomaterials

## Abstract

Corn cob is an agricultural byproduct that produces an estimated waste burden in the thousands of tons annually, but it is also a good source of xylan, an important bioactive polysaccharide. Silver nanoparticles containing xylan (nanoxylan) were produced using an environmentally friendly synthesis method. To do this, we extracted xylan from corn cobs using an ultrasound technique, which was confirmed by both chemical and NMR analyses. This xylan contained xylose, glucose, arabinose, galactose, mannose, and glucuronic acid in a molar ratio of 50:21:14:9:2.5:2.5, respectively. Nanoxylan synthesis was analyzed using UV–vis spectroscopy at kmax = 469 nm and Fourier transform infrared spectroscopy (FT-IR), which confirmed the presence of both silver and xylan in the nanoxylan product. Dynamic light scattering (DLS) and atomic force microscopy (AFM) revealed that the nanoxylan particles were ~102.0 nm in size and spherical in shape, respectively. DLS also demonstrated that nanoxylan was stable for 12 months and coupled plasma optical emission spectrometry (ICP-OES) showed that the nanoxylan particles were 19% silver. Nanoxylan reduced *Leishmania amazonensis* promastigote viability with a half maximal inhibitory concentration (IC50) value of 25 μg/mL, while xylan alone showed no effective. Additionally, nanoxylan exhibited antifungal activity against *Candida albicans* (MIC = 7.5 μg/mL), *C. parapsilosis* (MIC = 7.5 μg/mL), and *Cryptococcus neoformans* (MIC = 7.5 μg/mL). Taken together, these data suggest that it is possible to synthesize silver nanoparticles using xylan and that these nanoxylan exert improved antileishmanial and antifungal activities when compared to the untreated polysaccharide or silver nitrate used for their synthesis. Thus, nanoxylan may represent a promising new class of antiparasitic agents for use in the treatment of these microorganisms.

## 1. Introduction

Protozoa and fungi, despite being two distinct groups within the classification of life, have something in common; some representatives of both groups cause infections in other living organisms. Within protozoans, *Leishmania* species are the most likely to cause infection (Leishmaniasis). Leishmaniasis is one of the most prominent neglected infectious diseases in the world. It is endemic in 98 countries, and approximately 350 million people are susceptible to this disease [1], with an estimated 12 million infections worldwide [2]. There are several disease-causing fungi including organisms from both the *Candida* and *Cryptococcus* genera. These two genera cause candidiasis [3] and cryptococcosis [4], respectively. Factors such as immunosuppression and a lack of adequate treatment contribute to long hospital stays and high morbidity rates for people affected by these diseases [5,6].

Despite being caused by different pathogens, candidiasis, cryptococcosis, and leishmaniasis all share one major problem, that is, there have been several reports describing the emergence of treatment resistance in all three diseases [7,8,9]. Given the prevalence of these diseases and their emerging resistance, there has been a surge in the number of studies focused on developing novel therapeutics against these diseases [10,11], with the development of nanoparticles as a clear frontrunner [12,13].

Nanoparticles can be described as solid and stable colloids with sizes ranging from 10 to 1000 nm [14], and they can be produced using various materials including metals such as silver, gold, zinc, and copper [15].

Silver has long been known as a bactericidal agent. However, with the advent of antibiotics, its use for this purpose has been reduced, limited mainly to the treatment of burns [16] and some skin or subcutaneous tissue infections, including perianal abscesses [17]. Recently, several microorganisms, including bacteria, protozoa, and fungi, have been shown to exhibit resistance to frontline therapeutics [18], thus driving the search for new antimicrobial agents as well as novel strategies to potentiate the action of the existing ones.

One way to increase the action of silver particles is to apply it in its nanoparticulate form [19]. Silver nanoparticles can be formed exclusively from silver [20] or via the combination of silver and one or more organic components [21]. In this case, the organic molecules reduce the silver particles creating an association, often using stable bonds, to produce the silver nanoparticles. These nanoparticles may have important biological activities, including antiviral activity against the herpes simplex virus type 2 [22], anti-inflammatory activity [23], and antiangiogenic activity [24].

There are several methods for silver nanoparticle synthesis, most of which can be described as multi-step processes that involve the use of more than one type of chemical compound [25]. Since these methods are generally harmful to the environment, as they generate toxic waste, many efforts have been made to develop synthesis protocols that use chemicals with low toxicity and/or that generate little to no residue, in an effort to reduce environmental impact. Such approaches are known as green synthesis or environmentally friendly methods. Green synthesis processes are gaining traction in nanotechnology as they use fewer polluting solvents and reducing agents. In this type of synthesis, water is used as a solvent, which guarantees a greater biocompatibility than the traditional organic solvents, often improving biological functionality [26].

Many studies have shown that polysaccharides meet the standards prescribed for green synthesis as they are low-toxicity molecules with excellent biocompatibility [27]. However, several polysaccharides have not been investigated in the synthesis of nanoparticles, particularly in the production of silver nanoparticles. One of these polysaccharides is corn cob xylan. In 2019–2020, approximately 1114.75 million tons of corn will be produced worldwide, [28] and in most cases, there will be tons of underutilized corn waste. However, this waste from the agrochemical industry may be an important resource in the production of xylan, which can be obtained easily and in great quantities using a variety of extraction methods [29].

In a previous study, we extracted a large amount of xylan from corn cob using an environmentally friendly extraction method and showed that this polysaccharide presented with different pharmacological properties [30,31]. However, the ability of this polysaccharide to act as a bioreactor in the synthesis of silver nanoparticles has not been evaluated. Thus, the objective of this work was to synthesize silver nanoparticles containing corn cob xylan (nanoxylan) and to evaluate their biological activity compared to regular xylan products.

## 2. Materials and Methods

### 2.1. Materials

Roswell Park Memorial Institute 1640 medium (RPMI 1640) and inactivated fetal bovine serum (BSF) were obtained from Gibco^®^, Life Technologies (Carlsbad, CA, USA). Prozima Prolav 750 was purchased from Prozyn Biosolutions (São Paulo, SP, Brazil). Ethylenediaminetetraacetic acid (EDTA), l-fucose, d-xylose, d-galactose, d-mannose, d-glucose, d-arabinose, d-rhamnose, d-glucuronic acid, N-d-acetylglucosamine, d-galactosamine, d-glucosamine, and silver nitrate were all obtained from Sigma Aldrich Co. (St. Louis, MO, USA). The other solvents and chemical reagents used in this study were of analytical grade.

### 2.2. Strains of Leishmania Amazonensis

*L. amazonensis* strains (MHOM/BR/73/M2269) were collected and identified using monoclonal antibodies and isoenzymes at the Evandro Chagas Institute (Belém, PA, Brazil). All strains were cultured in the Pathology of Infectious Diseases Laboratory at the Medical School of the University of São Paulo (São Paulo, Brazil).

### 2.3. Extraction of Xylan from Corn Cobs

Fresh ears of corn were bought at a local market and cleaned, and then the kernels were completely removed. The resulting corn cob was cut into small pieces, dried, and ground until turning into flour. To eliminate lipids and pigments, 200 mL of ethanol was added to 10 g of flour and this process was repeated until the pigments were not visible in the alcohol. This material was then used for xylan extraction under the following conditions: every 1 g of corn flour was supplemented with 25 mL of NaOH (1.8 M) and the solution was then subjected to ultrasonic waves (200 W) for 30 min at 5-min intervals. The entire procedure was performed at 60 °C. This suspension was then centrifuged (10,000× *g* for 15 min) to separate the soluble portion (containing xylan). All collection was sanctioned under the authorization given by the Brazilian National Management System Genetic Heritage and Associated Traditional Knowledge SISGEN n° AA90285.

### 2.4. NMR Spectroscopy

Heteronuclear single quantum coherence spectroscopy (HSQC) was performed using an Avance III HD Bruker 400 MHz spectrometer (Bruker, Billerica, MA, USA) equipped with a 5-mm broadband inverse probe at 30 °C. Xylan (20 mg) was dissolved in 500 μL of 99% (*v/v*) deuterium oxide (Cambridge Isotope Laboratory, Cambridge, MA, USA) and the chemical shifts in ^1^H and ^13^C are expressed in δ (ppm) relative to trimethylsilylpropionate (TMSP) which was used as an internal standard (δ = 0 ppm). The spectra were analyzed using TopSpin Bruker software version 3.2pl6 (Bruker Co., Billerica, MA, USA).

### 2.5. Determination of the Chemical Composition of the Sample

#### 2.5.1. Quantification of Sugars and Proteins

The amount of total sugars was determined by the phenol/sulfuric acid method [31], using xylose as the standard. The absorbance at 490 nm was determined using a microplate reader (Epoch™ Microplate Spectrophotometer, Winooski, VT, USA) and the degree of protein contamination was evaluated using Coomassie Brilliant Blue R-250 (Sigma Aldrich Co., St. Louis, MO, USA) at 595 nm [32]. Bovine serum albumin was used as the standard.

#### 2.5.2. Phenolic Compounds

The phenolic compounds were quantitatively evaluated using the Folin–Ciocalteu colorimetric method, with gallic acid as the standard, and the absorbance at 765 nm was determined using a microplate reader (Epoch™ Microplate Spectrophotometer) [30].

#### 2.5.3. Determination the Monosaccharide Composition of Xylan

Hydrolysis was performed using the conditions described by Melo-Silveira et al. [30] (2.0 M HCl, 2 h). After hydrolysis, sugar composition analysis was performed using the LaChrom Elite^®^ VWR-Hitachi HPLC System (Hitachi Ltd., Ibaraki, Japan) with an L-2490 refractive index detector (Hitachi Ltd., Ibaraki, Japan). A LichroCART^®^ 250-4 column (250 mm × 40 mm) packed with Lichrospher^®^ 100 NH2 (5 μm) was coupled to the system. A total of 0.2 mg of the sample was used, and the analysis took 25 min. The following monosaccharides were analyzed as references: arabinose, galactose, glucose, glucuronic acid, fructose, fucose, mannose, N-acetylglucosamine, galactosamine, glucosamine, rhamnose, and xylose.

### 2.6. Nanoxylan Synthesis

Silver nanoxylans were produced using a green synthesis procedure similar to that described by Dipankar and Murugan [33] using corn cob xylan as a bioreactor. The synthesis of nanoxylans was performed following the mixing of a 1.0 mM silver nitrate solution with a 10 mg/mL xylan solution (1:9 *v/v*). This mixture was homogenized in a round bottom flask, protected from light using aluminum foil, and subjected to stirring. After 24 h, the suspension was centrifuged at 10,000× *g* for 15 min at 25 °C, and the precipitate was collected, lyophilized, and protected from light in a desiccator.

### 2.7. Xylan Infrared Spectroscopy

Infrared spectra for the xylan and nanoxylan were obtained using a Fourier transform infrared spectrometer (IRAffinity-1, Shimadzu Corp., Kyoto, Japan) equipped with the IRsolution1.20 software (Shimadzu Corp., Kyoto, Japan). The samples were analyzed as tablets containing KBr. The analysis frequency range was 4000–400 cm^−1^ and three independent analyses were performed.

### 2.8. Characterization of Nanoparticles

#### 2.8.1. Spectroscopy

The reduction in silver (I) by xylan from corn cob and the formation of silver nanoparticles coated with xylan was accompanied by a color change in the solution. This change was monitored using electronic spectroscopy in the ultraviolet and visible ranges. Screenings from 350 to 600 nm were carried out for each nanoxylan solution using a DR 5000 (Hitachi Ltd., Ibaraki, Japan) spectrophotometer.

#### 2.8.2. Dynamic Light Scattering

The nanoparticle diameter was determined using photon correlation spectroscopy performed on a Brookhaven 90 Plus DLS (Brookhaven Instruments Corporation, New York, NY, USA). Nanoxylans (0.25 mg/mL) in water were analyzed in three independent experiments subjected to triplicate analyses, and all the values were reported relative to the mean. To evaluate the stability of the nanoparticles, a 0.25 mg/mL suspension was prepared and stored at 25 °C for 12 months, and was evaluated every thirty days as described above.

#### 2.8.3. Quantification of the Silver in the Nanoxylan Particles

##### Digestion of the Sample

To digest the sample, 100 mg of nanoxylan was placed in Teflon containers with 7 mL of 65% nitric acid, previously purified by sub-boiling distillation (Berghof, Eningen, Germany), and 1 mL of hydrogen peroxide 30% (Merck, Darmstadt, Germany). The containers were then closed and placed in a microwave digester (Start D, Milestone, Italy) and the digestion was performed in 6 stages at 1100 W: (1) 5 min at 70 °C; (2) 2 min at 70 °C; (3) 3 min at 120 °C; (4) 2 min at 120 °C; (5) 10 min at 170 °C; (6) 15 min at 170 °C; and then ventilated for 30 min before removing the rotor from the microwave. The digested sample was placed in 15 mL of deionized water and filtered prior to further analysis (0.45-μm membrane). Analyses were carried out in duplicate and controls were performed following the procedure, in the absence of the sample.

##### Determination of Silver Content

We used inductively coupled plasma emission spectrometry (ICP-OES 5100 VDV, Agilent Technologies, Tokyo, Japan) to evaluate the silver content in each nanoparticle. ICP-OES was carried out in the axial view and equipped with a 27-MHz radio frequency (RF) source, a simultaneous optical detector, a peristaltic pump, a double-step cyclonic nebulization chamber, a 1.8-mm quartz torch, and a sea-spray glass nebulizer. The system used 99.996% liquid argon (White Martins, SP, Brazil) as the plasma gas. The operating conditions of the ICP-OES equipment were as follows: plasma power, 1.5 kW; argon flow rate, 12.0 L/min; auxiliary argon flow rate, 1.0 L/min; mist flow rate, 0.70 L/min; number of replicates, 3; stabilization and reading time, 15 s; and wavelength, 328.068 nm.

The analytical curve for silver composition was prepared by diluting a standard reference solution of 1000 mg/L in the range between 2.5 and 100 μg/L (r = 0.9999) in 5% (*v/v*) nitric acid solution prepared from the 65% distilled acid created in the sub-boiling system (Distillacid, Berghof, Eningen, Germany). Samples were successively diluted as: (i) 0.1 mL/10 mL, and (ii) 0.2 mL/10 mL. Thereafter, evaluation was performed.

#### 2.8.4. Atomic Force Microscopy (AFM), Scanning Electron Microscopy (SEM), Energy Dispersive X-ray Spectroscopy (EDS) and X-ray Diffraction Analysis

AFM images were obtained using a Scanning Probe Microscope SPM-9700 (Shimadzu Corp., Kyoto, Japan), and EDS analysis were performed by a Scanning Electron Microscope TM-3000 (Hitachi, Ibaraki, Japan) at the Scanning Electron Microscopy Laboratory (LABMEV) at the Materials Engineering Department (DEMat) of the Federal University of Rio Grande do Norte (UFRN). Three images were taken for each nanoxylan evaluation.

AFM analysis were made with dry nanoxylan that was dried on a glass cover slip. Ten microliters of the nanoxylan suspension (0.5 mg/mL) was dried onto carbon tape and submitted to EDS analysis.

Nanoxylan was processed and analyzed by SEM (Shimadzu Electron microscope, model SSX550; Shimadzu Corp., Kyoto, Japan). Briefly, 20 μL of each nanoxylan (0.5 mg/mL) sample was loaded onto a carbon-coated copper grid without gold coating and air-dried for 10 min under vacuum. The grid chamber was then placed in the SEM room and incubated in the dark at 10–20 °C for 2 h. Representative images of each of the three independent experiments are shown.

Powder characterization was performed using X-ray diffraction (XRD) on a Shimadzu XRD-7000 diffractometer (Cu Kα radiation, with 40 kV and 40 mA). The diffraction patterns were obtained in the angular range 20 ≤ 2θ ≤ 80° using the step-scanning mode (0.021/step, 2 s/step).

#### 2.8.5. Infrared Spectroscopy of Nanoxylan

This was performed as described above (2.7).

### 2.9. Evaluation of the Fe^2+^-Chelating Capacity of Xylan and Nanoxylan

The iron-chelating activity of xylan and the nanoxylan particles was determined as per previously described methods [30]. Briefly, the reaction mixture consisted of xylan or nanoxylan (0.001 to 0.1 mg/mL), FeCl_2_ (2 mM), and ferrozine (5 mM). After 10 min of incubation at 37 °C, the absorbance at 562 nm was determined using a microplate reader (Epoch™ Microplate Spectrophotometer). A reaction solution without the test sample was used as a blank and EDTA was used as a positive control.

### 2.10. Determination of Antileishmanial Activity

*L. Amazonensis* strains were cultured in vivo using 8-week-old BALB/c mice to maintain their infectivity. For all the evaluations, the parasites were extracted from the paw of the mice, isolated, and cultured at 25 °C in RPMI 1640 medium, supplemented with 10% fetal bovine serum (FBS)-inactivated, 10 μg/mL gentamicin, and 100 IU/mL penicillin. The parasite was used in its promastigote form isolated during the stationary phase of growth.

We used the protocol described by Passero et al. [34] to determine the antileishmanial activity of our nanoxylan particles. Stock solutions (1 mg/mL) were prepared in RPMI 1640 medium containing xylan or nanoxylan. These samples were then used at 5.0; 15.0; 25.0; 50.0, and 100.0 μg/mL. These samples were placed in 96-well microplates together with the *L. amazonensis* strains in their promastigote form (3 × 10^7^ promastigote/mL) and incubated at 25 °C for 24 h. *L. amazonensis* viability was then evaluated using an 3-(4,5-dimethylthiazol-2-yl)-2,5-diphenyltetrazolium bromide (MTT) assay.

The microplates containing samples were centrifuged (4000× *g*, 10 °C, 10 min) and the supernatant was carefully removed. The wells were washed twice with phosphate buffered saline PBS (0.1 M, pH 7.6), and then 50 μL of the MTT solution (4 mg/mL) was added. Plates were incubated at room temperature for 4 h and then 50 μL of 10% SDS was added. Plates were then incubated for an additional 18 h at 25 °C and then read at 595 nm. Leishmanial viability was then calculated using the following formula:

% MTT Reduction = Abs Sample (570 nm) × 100/Abs Control (570 nm)
(1)

### 2.11. Determination of Antifungal Activity

We used the Clinical & Laboratory Standards Institute - CLSI protocols (documents M27-A3 and M27-S3) [35,36], with minor modifications, to determine the minimum inhibitory concentration (MIC) and antifungal activity of the compounds in this study. These experiments were carried out using three different yeasts, two *Candida* species (*Candida albicans* (regional isolate) and *Candida parapsilosis* (ATCC22019–control test)), and one *Cryptococcus neoformans* isolate, provided by the Laboratory of Medical Mycology, from the Institute of Tropical Medicine at Rio Grande do Norte State, IMT-RN, part of the Federal University of Rio Grande do Norte, UFRN. Antifungal tests were performed using the microdilution protocol in 96-well plates. The concentrations of xylan and nanoxylan ranged from 1000 μg/mL to 1.95 μg/mL. Amphotericin B (16–0.313 μg/mL) and AgNO_3_ (200–0.39 μg/mL) were used as controls. Both the sterility control (medium plus drug without fungi) and the growth control (medium plus fungi without drugs) plates were incubated at 35 °C. MIC values were defined as the lowest concentration that caused 100% inhibition of growth compared to that of the drug-free control after the maximum time to incubation, which was 48 h for *Candida* and 72 h for *Cryptococcus*.

### 2.12. Mitochondrial Reduction in MTT Assay

The cytotoxicity of xylan and nanoxylan was evaluated using the mitochondrial reduction in MTT. Briefly, this assay evaluates the cellular capacity to reduce 3-(4,5-dimethylthiazol-2-yl)-2,5-diphenyltetrazolium bromide (MTT) to formazan, thereby indicating that these cells are metabolically active. A total of 1 × 10^3^ 3T3 cells were incubated in Dulbecco’s modified Eagle medium (DMEM) (control), DMEM with xylan (1.95, 3.91, 7.81, 15.63, 31.25, 62.5, 125, 250, 500 and 1000 µg/mL) or DMEM with nanoxylan (1.95, 3.91, 7.81, 15.63, 31.25, 62.5, 125, 250, 500 and 1000 µg/mL) at 37 °C for 24 h. The medium was then removed and MTT in DMEM was added and incubated for 4 h. Then, the medium was aspirated, and the formazan crystals were dissolved in 96% ethanol. Absorbance was measured at 570 nm.

### 2.13. Statistical Analysis

Data are expressed as the mean ± standard deviation. Data were collected in triplicate and analyzed using analysis of variance (ANOVA). The Student–Newman–Keuls test (*p* < 0.05) was used to determine significant differences among the samples using GraphPad Prism version 5.0, 2014 (La Jolla, CA, USA).

## 3. Results

### 3.1. Chemical Analysis of Corn Cob Xylan

The extraction process used in this study resulted in a conversion efficiency of 35.0% ± 2.0% xylan relative to the mass of corn cob meal used.

Table 1 shows the results obtained from the chemical analysis of the xylan. Xylose (50%) was the major polysaccharide in these extracts but there were also relevant quantities of glucose and arabinose (>10%). There were also several less significant contributors (less than 10%), including galactose, mannose, and glucuronic acid. Additionally, the xylan extracts presented with low levels of proteins and phenolic compounds. For comparison, Table 1 also summarizes the chemical composition of the xylan produced by Melo-Silveira et al. [30].

### 3.2. NMR Analysis

**Figure 1:**
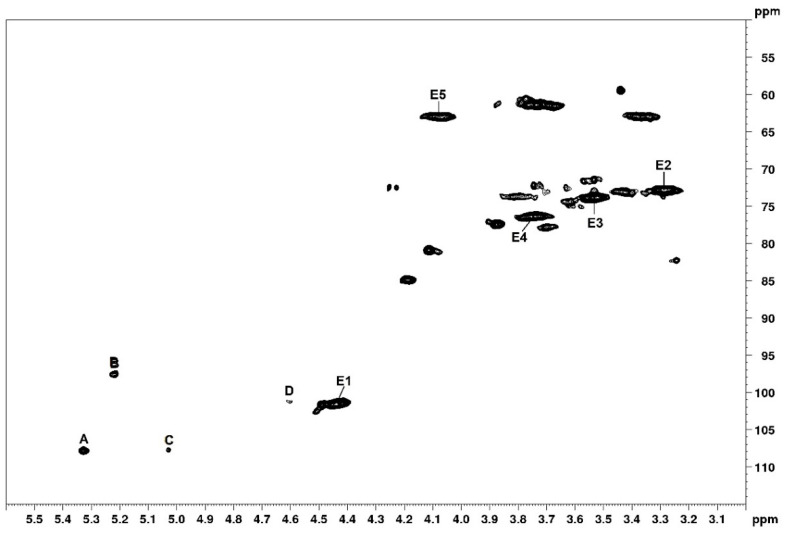
HSQC spectra of xylan from corn cob.

Two-dimensional NMR analysis was carried out to confirm that it was xylan that was extracted from the corn cob meal. Xylan’s HSQC spectrum (Figure 1) includes five major anomeric 1H/13C cross-peaks named A, B, C, D, and E. Unit E shows an intense anomeric correlation at 4.44/101.6 ppm, thereby confirming the presence of higher amounts of monosaccharide residues. Additionally, signals A and B have similar intensities. Signals C and D have similar intensities.

### 3.3. Synthesis of Nanoxylan

**Figure 2:**
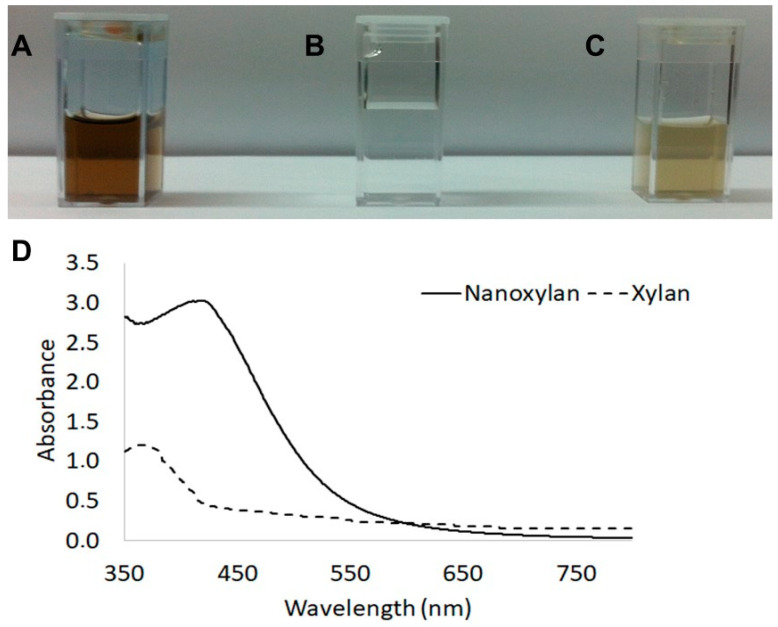
Nanoxylan suspension (**A**), silver nitrate 1 mM solution (**B**), and xylan solution (10 mg/mL) (**C**). The darker color in the nanoxylan suspension is related to the reduction in the silver particles. UV–vis absorption spectrum of the nanoxylan suspension and xylan solution (**D**).

Figure 2 shows an image of the solutions used in this process. Figure 2A shows the suspension of silver nanoparticles produced using xylan, 2B shows the silver nitrate solution, and 2C shows the xylan solution. It should be noted that the nanoparticle suspension (2A) has a darker color than the other two solutions.

Thus, the nanoparticle suspensions were evaluated by UV–vis spectroscopy. Figure 2D shows the light absorption scanning spectrum for the nanoparticle suspension and xylan solution. We observed absorbance values of around 2.900 in the region close to 350 nm of the nanoxylan curve; however, these values begin to increase in the region of 400 nm and reach a maximum absorption in the 3.100 nm region. It is worth noting that when the xylan solution was analyzed, the highest absorption peak was observed at 360 nm.

A total of 40 mg of the material was isolated after lyophilization of nanoxylan suspensions, which gave this process a yield of 36.6% in relation to the amount of starting materials used (100 mg of xylan and 10 mg of silver nitrate).

### 3.4. Characterization of Nanoxylan

**Figure 3:**
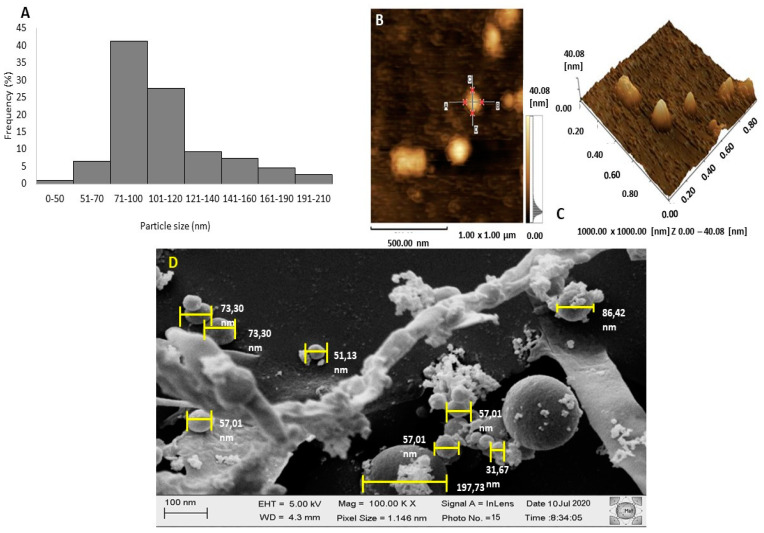
Physical attributes of the nanoxylan particles. (**A**) Size dispersion histogram obtained using dynamic light scattering (DLS). (**B**,**C**) AFM images of the nanoxylan particles. (**B**) Nanoxylan in two dimensions, with two planes drawn in red; (**C**) nanoxylan in three dimensions; (**D**) SEM image of the nanoxylan particles.

The mean volume hydrodynamic size of these particles in water was measured by DLS and shown to be 101.4 ± 2.7 nm (Figure 3A). The average size of nanoxylan was also evaluated using AFM and shown to be approximately 40 nm. Additionally, AFM also suggested that the nanoxylan particles had a predominantly rounded/spherical shape (Figure 3B,C). The SEM images of the nanoxylan suspensions provide information on the morphology and size of the nanoparticles (Figure 3D). The particles were predominantly spherical in shape, aggregated, and had an average size of 60.6 ± 27 nm, although some large nanoparticles were visible. This discrepancy can be easily explained by the differences in the sample preparation process used for SEM and AFM analyses, where the particles are dehydrated, and DLS which is performed in aqueous solutions.

**Figure 4:**
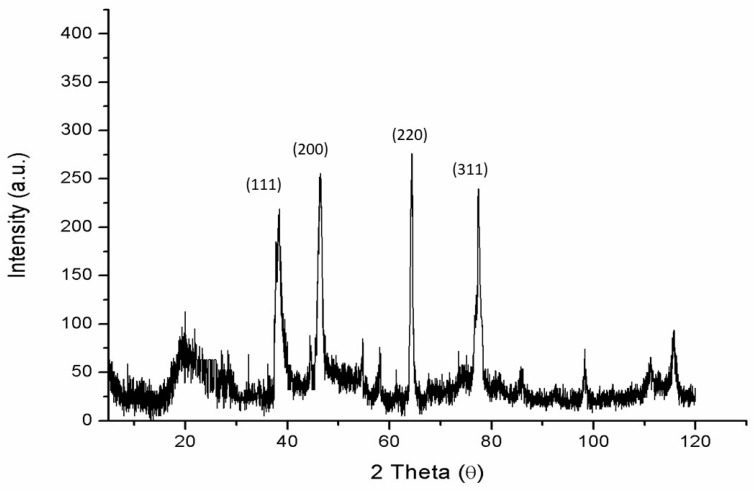
XRD patterns of nanoxylan.

The XRD patterns for these nanoxylan particles are shown in Figure 4. The peak positions match well with those of metallic silver and show no observable impurities.

**Figure 5:**
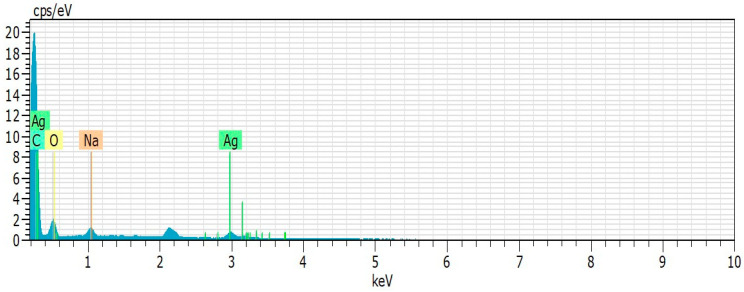
Energy dispersive X-ray spectroscopy (EDS) spectrum of nanoxylan.

EDS analysis (Table 2 and Figure 5) shows the elements on the surface of nanoxylan. Carbon, oxygen, silver, and sodium were the main elements. As the particles were coated with gold for EDS analysis, a peak situated at the binding energy of 2.1 keV belonging to Au was observed. The peaks observed at 0.18 and 3.0 keV correspond to the binding energies of silver. No peaks of other impurities were detected, indicating that the silver nanoparticle sample contains pure silver, with no oxide.

We also used ICP-OES to determine the amount of silver in the nanoxylan particles and found that silver contributed to 19% of their total dry weight. In addition, phenol/sulfuric acid method [31] showed sugar (80%) as the other major component found in nanoxylan.

Based on these results, it was possible to infer that the nanoxylan particles were formed following interactions between the corn xylan and silver particles.

**Figure 6:**
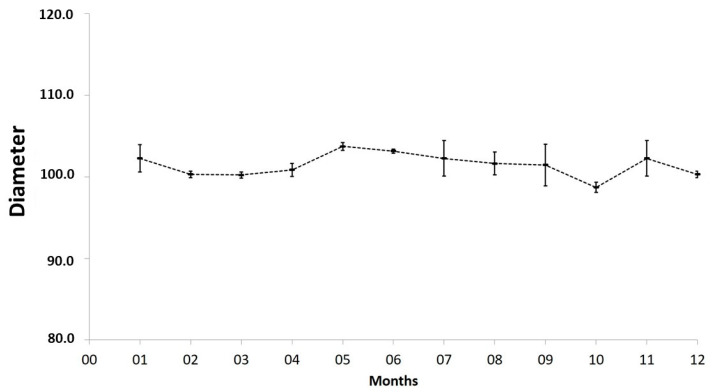
Average size of the nanoxylan particles evaluated over a 12-month period.

To verify the stability of these particles, their size was periodically measured over a 12-month period, and the results are shown in Figure 6. The mean size of nanoxylan was 102.3 ± 1.70 nm in month one and 100.3 ± 0.40 nm in month 12, but these were not statistically significant differences, suggesting that these nanoxylans were reasonably stable.

**Figure 7:**
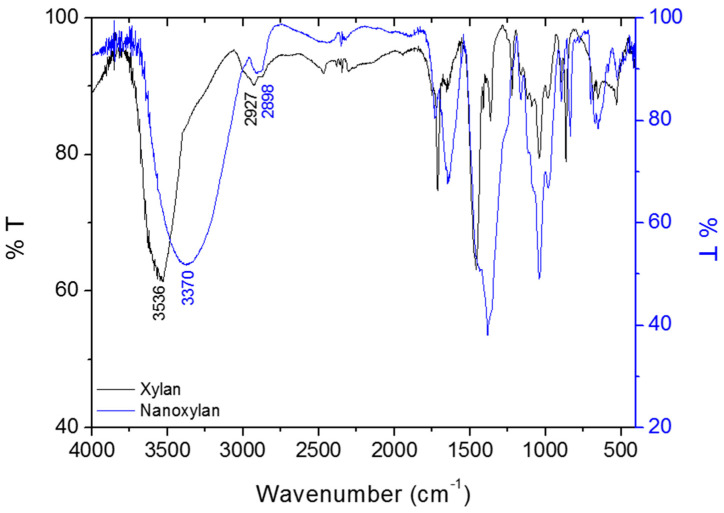
Overlapping infrared spectra for xylan (in black) and nanoxylan (in blue) between 4000 and 400 cm^−1^.

The infrared spectra for the xylan and nanoxylan are shown in Figure 7. The vibrational modes observed at 3536 and 3370 cm^−1^ can be attributed to the OH stretch present in xylan and nanoxylan, respectively.

It is important to emphasize that a free O-H stretch appears at higher frequencies because greater energy is required to create a vibration. Sp3 C-H stretches were confirmed with vibrations at 2927 cm^−1^ in the xylan mixtures and at 2898 cm^−1^ in the nanoxylan suspensions.

**Figure 8:**
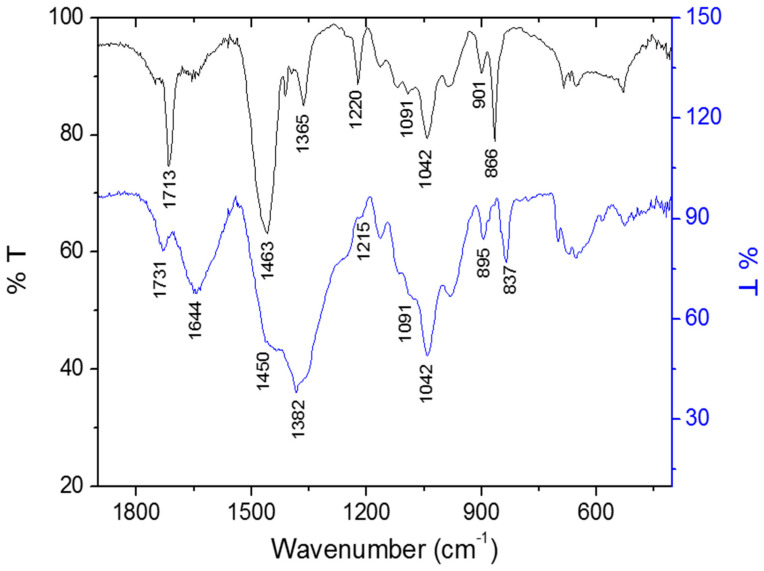
Overlapping infrared spectra of xylan (black) and nanoxylan (blue) in the 1900 to 400 cm^−1^ regions.

In Figure 8, we show the spectral pattern of the samples between 1900 and 400 cm^−1^ where it is possible to verify some of the differences in the vibration frequencies between nanoxylan and xylan. The bands observed at 1713 cm^−1^ (xylan) and 1731 cm^−1^ (nanoxylan) can be attributed to the C = O stretching vibration of the carboxyl groups found in uronic acid. The displacement of these bands is likely due to the addition of the silver particles. Additionally, the intensity of the 1731 cm^−1^ band decreases considerably in the presence of silver. Finally, the 1644 cm^−1^ vibrational frequency present in the nanoparticle spectrum can be attributed to absorbed water.

The bands around 1400 cm^−1^ correspond to the reduced silver particles. However, when we analyzed the nanoxylan spectrum, we found that the region around 1400 cm^−1^ was quite broad, thereby indicating an overlap between this vibrational mode and those of other groups.

The bands observed in the region of 1463–1220 cm^−1^ (xylan) and 1450–1215 cm^−1^ (nanoxylan) are linked to the angular deformation of the CH groups and the stretching vibration of CO, respectively. In contrast, the vibrational modes at 1091 cm^−1^ and 1042 cm^−1^ are related to the glycosidic ring, the angular deformation in C-O-H, and the axial deformation in C-O and C-O-C. It is also worth mentioning that the bands at 901 cm^−1^ (xylan) and 895 cm^−1^ (nanoxylan) can be attributed to the angular deformation of the anomeric β-glycosidic carbon bond between the monosaccharides. The vibrations at 866 cm^−1^ (xylan) and 837 cm^−1^ (nanoxylan) correspond to the presence of the furan ring.

### 3.5. Determination of the Iron-Chelating Activity of Xylan and Nanoxylan

**Figure 9:**
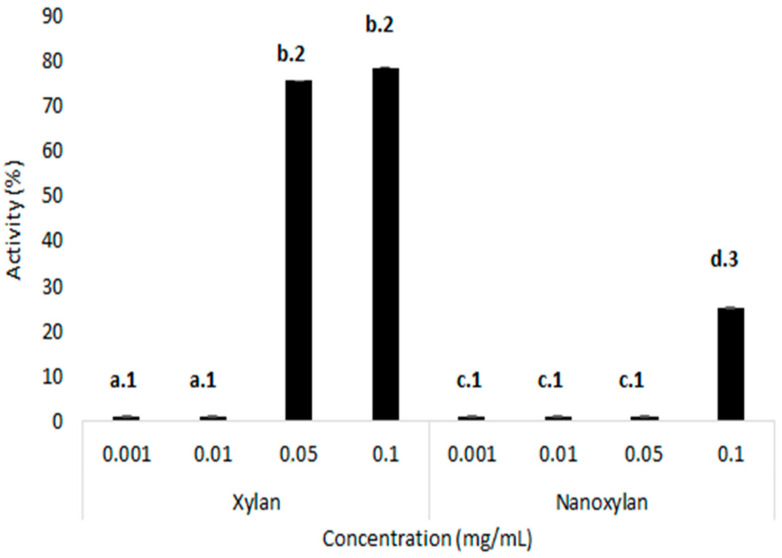
Iron-chelating activity of xylan and nanoxylan. Different letters (a, b, c, and d) represent statistically significant differences between the same concentration of xylan and nanoxylan. Different numbers (1, 2, and 3) represent statistically significant differences between different concentrations (0.001; 0.01; 0.5; 0.1 mg/mL) of xylan and nanoxylan (0.001; 0.01; 0.05; 0.1 mg/mL). *p* < 0.01.

Figure 9 shows the iron chelation activity of xylan and nanoxylan. Xylan has a higher iron-chelating activity than nanoxylan, where, at a concentration of 0.05 mg/mL, it can chelate approximately 75% of the available iron. Additionally, this activity remained constant even with an increased concentration. Nanoxylan only exhibited chelating activity at the highest concentration (0.01 mg/mL), when it was only able to chelate approximately 20% of the available iron.

### 3.6. Evaluation of Antileishmanial Activity

**Figure 10:**
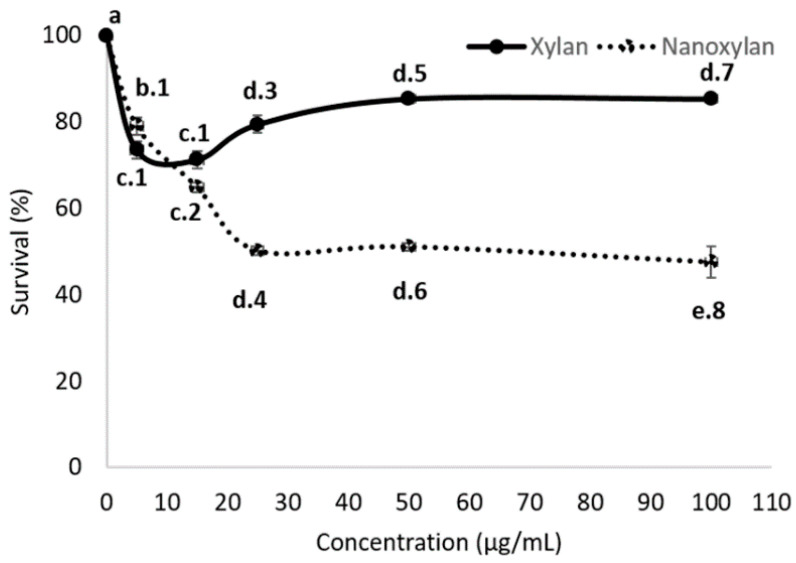
Antileishmanial activity of xylan and nanoxylans. Xylan and nanoxylan were evaluated at 0, 5, 15, 25, 50, and 100 μg/mL, respectively. Different letters (a, b, c, d, and e) represent statistically significant differences between different concentrations of xylan or nanoxylan (*p* < 0.05). Different numbers (1, 2, 3, 4, 5, 6, 7, and 8) represent statistically significant differences between the same concentration of xylan and nanoxylan (*p* < 0.05).

*L. amazonensis* promastigotes were treated with xylan and nanoxylan and the results are shown in Figure 10. Xylan exhibited low antiparasitic activity, thus reaching a maximum inhibition of approximately 22% of the parasites when tested at a concentration of 5 μg/mL. It is worth noting that increasing the concentration to 100 μg/mL did not change this activity. On the other hand, a low rate of parasite survival was especially noticeable on exposure to nanoparticle concentrations from 20 μg/mL to 100 μg/mL. The effect of silver nitrate was also evaluated, however, it (from 2.0 to 30 μg/mL) did not show an effect on parasite survival. It is noteworthy that the amount of silver nitrate used contained 30 μg/mL of silver, which is 1.5 times greater than that present in the 100 μg/mL of nanoxylan.

### 3.7. Antifungal Activity

The CLSI protocol was used to evaluate the minimal inhibitory concentration (MIC) for xylan and nanoxylan in the treatment of fungal growth, with the final MIC data recorded after 48 h for *Candida* and 72 h for *Cryptococcus.* However, since no significant differences were observed between the *Cryptococcus* samples between 48 h and 72 h, Table 3 shows the results of the 48-h incubation. The minimal inhibitory concentration (MIC) for amphotericin B (control drug) was different for each yeast species, with *C. neoformans* being more susceptible (MIC = 0.125 µg/mL) than *C. albicans* (MIC = 0.5 µg/mL). The MIC for the silver nitrate was the same for all the samples (25 µg/mL). Xylan did not exhibit any inhibitory effect, and nanoxylan was shown to inhibit fungal growth for all the yeast strains evaluated in this study at an MIC of 7.8 µg/mL.

### 3.8. Mitochondrial Reduction in MTT

**Figure 11:**
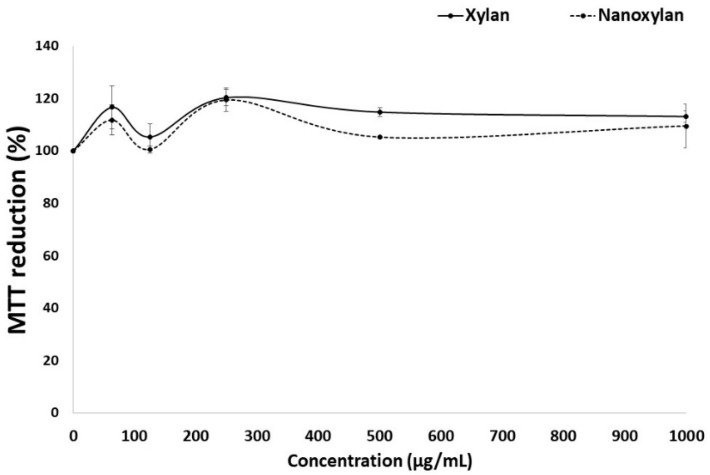
MTT reduction in cells treated with xylan or nanoxylan. There is no statistically significant difference between the different concentrations of xylan (*p* < 0.05).

3T3 cells were treated with nanoxylan and xylan to evaluate the ability to compare with the use of MTT. The results obtained are shown in Figure 11. The reduction in MTT was similar in the presence of xylan and nanoxylan, suggesting that the addition of the silver particles added cytotoxic properties to the nanoxylan particles.

## 4. Discussion

Our extraction methods yielded similar xylan quantities to those described by Melo-Silveira et al. [30], and more than those obtained by Ebringerová and colleagues (15%). [37]. However, the latter did not use an alkaline solution during the extraction process, which might have contributed to their lower yield.

The composition of the xylan products (monosaccharides) were similar to those described by Melo-Silveira and colleagues [30] (Table 1), thereby indicating a high degree of similarity between the evaluated materials. 2D-NMR analysis confirmed that xylan was mainly composed of xylose, glucose, and arabinose and xylan’s HSQC spectrum showed five major anomeric 1H/13C cross-peaks named A, B, C, D, and E. Units A and C correspond to →2)-α-L-Araf→1 and terminal α-L-arabinose residues, with anomeric correlations at 5.33/107.9 and 5.03/107.7 ppm, respectively [38]. Unit B had a cross-peak at 5.22/97.6 ppm, thereby confirming the presence of a terminal α-d-glucose [39]. Unit D had a signal at 4.60/101.3 ppm corresponding to β-d-galactose residues [40]. Finally, unit E exhibited an intense anomeric correlation at 4.44/101.6 ppm, thereby confirming the presence of a higher amount of xylose residues [41]. The xylan produced from corn cobs in the study reported by Melo-Silveira et al. [31] had a slightly different HSQC spectrum, but this might be because they performed their analyses at 70 °C and we performed ours at 30 °C. This reinforces the idea that temperature differences exert significant influence on the outcomes of NMR analysis. Nonetheless, our results are remarkably like the xylan NMR spectra reported in the literature, which suggests that the biological properties applied to this compound should also be applicable in this study [30,31].

Silver nanoparticles are synthesized using a reducing agent that promotes the reduction in Ag^+^ ions to Ag^0^, with sodium borohydride (NaBH_4_) and sodium citrate (Na_3_C_6_H_5_O_7_) being the most common in commercial applications. However, this in an environmentally unfriendly approach. Thus, in the present study, we described and evaluated the efficacy of a more environmentally friendly production system based on the work of Dipankar and Murugan [33]. In this method, reducers from natural sources can be used in place of the stringent organic solvents. These can include monosaccharides and plant extracts, which have become increasingly popular with the advent of green chemistry. Thus, we used xylan obtained from corn cob as the reducing agent in our study.

The darker color observed in the nanoparticle suspensions (Figure 2A) is the result of the reduction in silver ions [42]. This has been also specified as an indication of the formation of the nanoparticles, which was also confirmed by UV–vis spectroscopy [33]. Additionally, other authors, who synthesized silver nanoparticles [43], observed a similar increase in the spectra as shown in Figure 2D. Gurunathan and colleagues [44] suggested that this change was the result of the resonance in the plasmon surface produced by reduced silver.

The cytotoxicity of AgNPs can be considered as dependent on different kinds of AgNPs properties, such as nanoparticle size, shape, concentration, aggregation, and the type of coating materials [45]. Therefore, these nanoxylan parameters were evaluated here.

The mean size of our particles was like that observed by Chen and colleagues (102.00 nm) [46]. It is worth noting that these authors synthesized silver nanoparticles with fungal cell wall polysaccharides. This size is promising in light of the findings reported by Coradeghini et al. [47] and Elsabahy and Wooley [48], who have shown that nanoparticles with sizes between 20 and 200 nm in suspension are particularly useful in vivo because they are less likely to induce toxicity, as they do not incur as much membrane damage as that incurred by larger molecules.

As far as we know, all the silver nanoparticles synthesized in the presence of polysaccharides are round or spherical, including those formed from guan gum [43] and chitosan [49]. This is another positive aspect for these silver–polysaccharide nanoparticles, because there is a relationship between the shape and the cytotoxic characteristics of the nanoparticles, with the data showing that rounded forms are less cytotoxic than triangular, square, cubic or rectangular forms [50].

We found that nanoxylan was much more stable than other silver nanoparticles, since a majority of studies report that these nanoparticles exhibit stability between 25 and 90 days [45,51]. We were able to identify two other studies where the nanoparticles were stable for more than 90 days, wherein one study described the stability of fucan-conjugated nanoparticles [42,52]. Gunsolus et al. [53] suggested that the stability of AgNPs depended on the chemical composition of their coating agent. Nanoxylan showed size stability for several months, and this suggests that xylan acting as an organic capping agent prevents nanoparticle aggregation.

Nanoxylan did not show cytotoxicity under the conditions evaluated (from 50 to 1000 μg/mL) against 3T3 cells. This is an interesting fact, since other AgNPs showed cytotoxicity at much lower concentrations. The concentration resulting in 90% inhibition of 3T3 cell viability of uncoated AgNPs (>100 nm) was 9.59 μg/mL [54]. The green synthesized silver nanoparticles (136 ± 10.09 nm) were achieved using the aqueous extract of *Origanum vulgare* (Oregano). These AgNPs showed 100 μg/mL as LD50 against the A549 cell line [55].

It has been pointed out that the cytotoxicity of the nanoparticles comes from themselves and/or from the Ag^+^ released of the nanoparticle. AgNPs interact with cell surface receptors to activate intracellular pathways, which in turn increase oxidative stress. This fact leads to cellular damage, including nuclear DNA, inducing inhibition of cell proliferation and even cell death [56]. The surface of AgNPS undergoes oxidation in both the extra and intracellular medium, and this leads to the release of Ag^0^ and Ag^+^. This leads to the accumulation of ionic silver inside the cell, and, consequently, mitochondrial dysfunction and cell death [57].

Xylan is an exotic polysaccharide for the enzymatic system of a mammalian cell, which should hinder its degeneration by 3T3 cells. Therefore, we suggested that this may have protected silver present in nanoxylan from oxidation processes, leading to low ionic silver accumulation into 3T3 cells. In addition, the presence of xylan as a capping agent may have hindered the interaction of nanoxylan with cell surface receptors that activate pathways for the reactive species formation, and often, preventing the intracellular oxidative stress.

In summary, nanoxylan did not show cytotoxicity because it has a less toxic size and shape for mammalian cells, because it has a low capacity to aggregate, and because the presence of xylan can decrease the formation of reactive species and the accumulation of silver ions in the intracellular environment. However, further studies need to be carried out to better understand the role of xylan in decreasing the cytotoxicity of AgNPs.

The amount of silver in nanoxylan corresponds to 19% of its dry weight. There are a few studies that have determined the amount of silver in these silver nanoparticles. In one study, silver constituted 7% of the nanoparticle [42]; in another study, silver was shown to contribute between 24.65% and 36.24% of the total dry weight [58]. This indicates that the silver content of nanoxylan is in the same range as other nanoparticles.

The values observed in the infrared spectra were consistent with data from the literature discussing polysaccharides and silver nanoparticles [59,60]. Additionally, the intensity of the 1731-cm^−1^ band decreases considerably in the presence of silver, which is an indirect confirmation of the interaction between the xylan and the silver particles [61].

The formation of AgNPs using natural polysaccharides as reducing agents makes it possible to obtain nanoparticles containing these polymers. Thus, the biological characteristics already described in the literature for both silver and polysaccharides in a single material promote synergism between these components [21]. With this in mind, we synthesized nanoxylan and evaluated its chelating capacity, but here, the combination of materials reduced the activity of the polysaccharide component, with xylan exhibiting greater chelating power than that exhibited by nanoxylan. These data suggest that iron ion chelation is entirely linked to the “original” form of the polysaccharide. The reduction in the chelating activity can be explained by the decrease in free carbonyl groups in the polysaccharide, as these are key moieties in the polysaccharide–silver interactions. This hypothesis is confirmed by the study reported by Andersen [62] which suggested that these carbonyl groups were important for iron chelation, and that their loss, even if only partial, significantly decreased the chelating capacity of the polysaccharide compounds.

Nanoxylan was the most potent antileishmanial agent, showing significantly more antimicrobial activity than xylan (Figure 10). As far as we know, this is the first study to evaluate the antileishmanial activity of corn cob xylan. Additionally, we did not find any other studies evaluating the antileishmanial activity of silver nanoparticles containing polysaccharides. However, the results obtained here were interesting because uncoated AgNPs (20–30 nm) inhibited promastigote viability by 40%, but only at high concentrations 100 μg/mL [63], which was significantly higher than our 25 μg/mL concentration for the nanoxylan particles. Other AgNPs had a greater effect on the viability of promastigote forms than nanoxylan, but these showed greater cytotoxicity. For example, regarding AgNPs synthesized with *Cuminum cyminum*, only 0.5 µg/mL of these AgNPs were needed to decrease the viability of promastigotes by 50%. However, these AgNPs (1.56 µg/mL) reduced the viability of J774 macrophages by 91% [64]. AgNPS synthesized with tannic acid showed a half maximal inhibitory concentration (IC50) of 6.96 µg/mL, but also at this concentration they decreased the viability of murine peritoneal macrophages by 70% [65].

Ginouvès et al. [66] determined the IC50 of several antileishmanial drugs against the *L. amazonensis* promastigote form, amphotericin B (IC50 = 8.35 µg/mL), meglumine antimoniate (IC50 = 9822.0 µg/mL), miltefosine (IC50 = 1.95 µg mL), and paromomycin (IC50 = 129.6 µg mL). As can be seen, only amphotericin B and miltefosine were more potent than nanoxylan. However, these drugs are toxic, and strains resistant to them have been reported. Additionally, the production of nanoxylan potentiated the antimicrobial activity of this polysaccharide and demonstrated a new use for xylan. Given these promising results, it would be interesting to evaluate the antileishmanial mechanism of these nanoxylan particles.

The antifungal assay, which tested three different yeast strains (Table 2), confirmed the increased antimicrobial activity of the nanoxylan particles. Amphotericin B was used as the positive control in this analysis, as it is the most common drug used in the clinical management of these infections [67], but growing resistance and severe side effects increase the demands for alternative therapeutics. Among these molecules, silver nanoparticles are known to penetrate microbial cells and affect membrane integrity, thereby leading to a disturbance of cellular permeability and respiration capacity, thus triggering cellular content release [68]. The use of AgNPs has already been shown to exert inhibitory effects on fungal growth in pathogenic yeasts *Candida* and *Cryptococcus* [69]. In our experiment, we observed that nanoxylan obtained an inhibitory concentration equivalent to approximately 63× the active concentration of amphotericin B. However, because it is a natural compound, nanoxylan may act in association with other antifungal drugs, which may potentiate the inhibitory action profile of these antifungal drugs or serve as a basis for other nanoparticle-bound natural compounds.

In both models studied here, nanoxylan was more effective against microorganisms than silver nitrate. Furthermore, xylan did not show microbicidal activity against *L. amazonensis* and fungi. Although the mechanisms of action of AgNPs as antifungal or antileishmanial agents has not been properly elucidated, it has been proposed that AgNPs can adhere to the surface of the cell and alter the transmembrane permeability, which would lead to cell death. Therefore, we propose that xylan, being a polysaccharide, should have been more easily recognized by the surface receptors of microorganisms and, therefore, it should have facilitated the interaction of nanoxylans with their surface, subsequently facilitating the microbicidal action of nanoxylan. We hope to verify this suggestion as soon as possible.

More than 337 million metric tons of corn cob are produced every year, and much of this production is wasted by the agricultural industry. These large quantities of corn cob are discarded in the wild and serve as a major source of pollution in various natural environments. To reduce this problem, it is important to find new uses for corn cobs. Additionally, bioactive molecules such as xylan are found in relatively high concentrations in corn cob. Therefore, we used corn cob as a source of xylan in this study and we hope that our results will encourage other groups to consider this waste product as a source for their xylan and other polysaccharide needs.

## 5. Conclusions

In this study we developed a simple eco-friendly method to produce xylan containing silver nanoparticles. These particles were stable for 12 months, had an average size of 102.0 nm, and exhibited a round shape. These nanoparticles were called nanoxylan and while they were a less potent antioxidant agent compared to xylan, they were more potent antileishmanial and antifungal agents than xylan or AgNO_3_, without inducing any cytotoxic effects in mammalian cells. Future studies should be designed to uncover the antileishmanial and antifungal mechanisms of nanoxylan.

## Figures and Tables

**Table 1:** Chemical composition of xylan from corn cobs.

Sample	Sugar (%)	Proteins (%)	Phenolic c. (%)	Molar Ratio ^a^ (%)					
				Xyl	Ara	Glu	Gal	Man	GlucA
Xylan	98.2 ± 1.9	1.05 ± 0.04	0.02 ± 0.01	50.0	16.0	21.0	8.0	2.5	2.5
Xylan ^b^	70	0.4	<0.01	50.0	15.0	20.0	10.0	2.5	2.5

Xyl: xylose; Ara: arabinose; Glu: glucose; Gal: galactose; Man: mannose; GlucA: glucuronic acid. ^a^ Analyzed by HPLC after acid hydrolysis at 100 °C for 2 h. ^b^ Data from Melo-Silveira et al. [30].

**Table 2:** Energy dispersive X-Ray spectroscopy elemental composition of nanoxylan.

	Carbon (%)	Oxygen (%)	Silver (%)	Sodium (%)
**Nanoxylan**	27.8	57.1	10.4	4.5

**Table 3:** Minimal inhibitory concentration (MIC) of Amp B (Amphotericin B), nanoxylan, xylan and AgNO_3_ against *Candida albicans*, *C. parapsilosis,* and *Cryptococcus neoformans* at 48 h of incubation.

	MIC (µg/mL)			
Yeasts	48 h			
	Amp B	Nanoxylan	Xylan	AgNO_3_
***Candida albicans***	0.5	7.8	Ni	25
***Candida parapsilosis***	0.25	7.8	Ni	25
***Cryptococcus neoformans***	0.125	7.8	Ni	25

Ni—no inhibition.
